# Supplementary material for: Dystrophy-associated caveolin-3 mutations reveal that caveolae couple IL6/STAT3 signaling with mechanosensing in human muscle cells
Source: Nat Commun. 2019 Apr 29;10:1974. doi: 10.1038/s41467-019-09405-5 (PMC6488599; doi:10.1038/s41467-019-09405-5)
Supplement: Supplementary file 2 — Reporting Summary [file 41467_2019_9405_MOESM2_ESM.pdf]

nature research

Corresponding author(s): Christophe LAMAZE, Cedric BLOUIN

Last updated by author(s): Jan 29, 2019

Reporting Summary

Nature Research wishes to improve the reproducibility of the work that we publish. This form provides structure for consistency and transparency in reporting. For further information on Nature Research policies, see Authors & Referees and the Editorial Policy Checklist.

Statistics

For all statistical analyses, confirm that the following items are present in the figure legend, table legend, main text, or Methods section.

n/a	Confirmed	
☐	☒	The exact sample size (n) for each experimental group/condition, given as a discrete number and unit of measurement
☐	☒	A statement on whether measurements were taken from distinct samples or whether the same sample was measured repeatedly
☐	☒	The statistical test(s) used AND whether they are one- or two-sided<br>*Only common tests should be described solely by name; describe more complex techniques in the Methods section.*
☐	☒	A description of all covariates tested
☒	☐	A description of any assumptions or corrections, such as tests of normality and adjustment for multiple comparisons
☐	☒	A full description of the statistical parameters including central tendency (e.g. means) or other basic estimates (e.g. regression coefficient) AND variation (e.g. standard deviation) or associated estimates of uncertainty (e.g. confidence intervals)
☐	☒	For null hypothesis testing, the test statistic (e.g. F, t, r) with confidence intervals, effect sizes, degrees of freedom and P value noted<br>*Give P values as exact values whenever suitable.*
☒	☐	For Bayesian analysis, information on the choice of priors and Markov chain Monte Carlo settings
☒	☐	For hierarchical and complex designs, identification of the appropriate level for tests and full reporting of outcomes
☐	☒	Estimates of effect sizes (e.g. Cohen's d, Pearson's r), indicating how they were calculated

Our web collection on statistics for biologists contains articles on many of the points above.

Software and code

Policy information about availability of computer code

Data collection: Image Lab (v5.2.1, ChemiDoc MP Imaging System, Bio-Rad) was used for chemiluminescence acquisition

Data analysis: Western Blot and microscope images were analyzed with ImageJ (v1.52, NIH)

For manuscripts utilizing custom algorithms or software that are central to the research but not yet described in published literature, software must be made available to editors/reviewers. We strongly encourage code deposition in a community repository (e.g. GitHub). See the Nature Research guidelines for submitting code & software for further information.

Data

Policy information about availability of data

All manuscripts must include a data availability statement. This statement should provide the following information, where applicable:
- Accession codes, unique identifiers, or web links for publicly available datasets
- A list of figures that have associated raw data
- A description of any restrictions on data availability

N/A

Field-specific reporting

Please select the one below that is the best fit for your research. If you are not sure, read the appropriate sections before making your selection.

☒ Life sciences ☐ Behavioural & social sciences ☐ Ecological, evolutionary & environmental sciences

For a reference copy of the document with all sections, see nature.com/documents/nr-reporting-summary-flat.pdf

Life sciences study design

All studies must disclose on these points even when the disclosure is negative.

Sample size	Experimental conditions (number of independent experiments, numbers of cells or fields per experiment...) were chosen according to standard procedures in cell biology research, and statistical significance was tested on the corresponding results.
Data exclusions	N/A
Replication	All replicates reported in the manuscript.
Randomization	Samples were not randomized for the experiments.<br>No randomization of mice. Mice analyzed were litter mates and sex-matched whenever possible.
Blinding	Investigators were not blinded to cell/mouse genotypes during experiments.

Reporting for specific materials, systems and methods

We require information from authors about some types of materials, experimental systems and methods used in many studies. Here, indicate whether each material, system or method listed is relevant to your study. If you are not sure if a list item applies to your research, read the appropriate section before selecting a response.

Materials & experimental systems

n/a	Involved in the study	
☐	☒	Antibodies
☐	☒	Eukaryotic cell lines
☒	☐	Palaeontology
☐	☒	Animals and other organisms
☒	☐	Human research participants
☒	☐	Clinical data

Methods

n/a	Involved in the study	
☒	☐	ChIP-seq
☒	☐	Flow cytometry
☒	☐	MRI-based neuroimaging

Antibodies

Antibodies used	Mouse anti-αTubulin (Sigma-Aldrich, clone B512, T5168, 1/1000 for WB); mouse anti-caveolin-3 (Santa Cruz, clone A3, sc-5310, 1/1000 for WB, 1/250 for IF); rabbit anti-caveolin-1 (Cell Signaling, 3238, 1/1000 for WB, 1/500 for IF); goat anti-GM130 (Santa Cruz, clone P-20, sc-16268, 1/50 for IF); mouse anti-MF20 (myosin 4) (kind gift of Vincent Mouly, 1/100 for WB, 1/20 for IF); mouse anti-STAT3 (Cell Signaling, clone 124H6, 9139, 1/1000 for WB); rabbit anti-pSTAT3 (Cell Signaling, clone D3A7, 9145, 1/1000 for WB, 1/75 for IF); Secondary antibodies conjugated to Alexa FITC, Cy3, Cy5 (1/200 for IF) or horseradish peroxidase (1/1000 for WB) (Beckman Coulter or Invitrogen).
Validation	Reported from the companies that provide the antibodies

Eukaryotic cell lines

Policy information about cell lines

Cell line source(s)	Platform for Immortalization of human cells of the Institute of Myology
Authentication	Myobank-AFM BB-0033-0012
Mycoplasma contamination	The cell lines used in this study were regularly tested for mycoplasma contamination. Only mycoplasma negative cells were used for experiments (colorimetric (MycoAlert™ PLUS Mycoplasma Detection Kit from Lonza) and/or PCR test (VenorGeM OneStep Mycoplasma Detection Kit from Minerva Biolabs)).
Commonly misidentified lines<br>(See ICLAC register)	N/A

Animals and other organisms

Policy information about studies involving animals; ARRIVE guidelines recommended for reporting animal research

Laboratory animals	immunodeficient mice, Rag2-/- Il2rb-/-, males, 2-to 3-month old

Wild animals	N/A
Field-collected samples	N/A
Ethics oversight	The protocol was approved by the Committee on the Ethics of Animal Experiments Charles Darwin N°5 (Protocol Number: 16903-2018092717023720).

Note that full information on the approval of the study protocol must also be provided in the manuscript.
