# Supplementary material for: Dystrophy-associated caveolin-3 mutations reveal that caveolae couple IL6/STAT3 signaling with mechanosensing in human muscle cells
Source: Nat Commun. 2019 Apr 29;10:1974. doi: 10.1038/s41467-019-09405-5 (PMC6488599; doi:10.1038/s41467-019-09405-5)
Supplement: Supplementary file 3 — Supplementary Information [file 41467_2019_9405_MOESM3_ESM.pdf]

Dystrophy-associated caveolin-3 mutations reveal that caveolae couple IL6/STAT3 signaling with mechanosensing in human muscle cells

Dewulf et al.

Supplementary Information

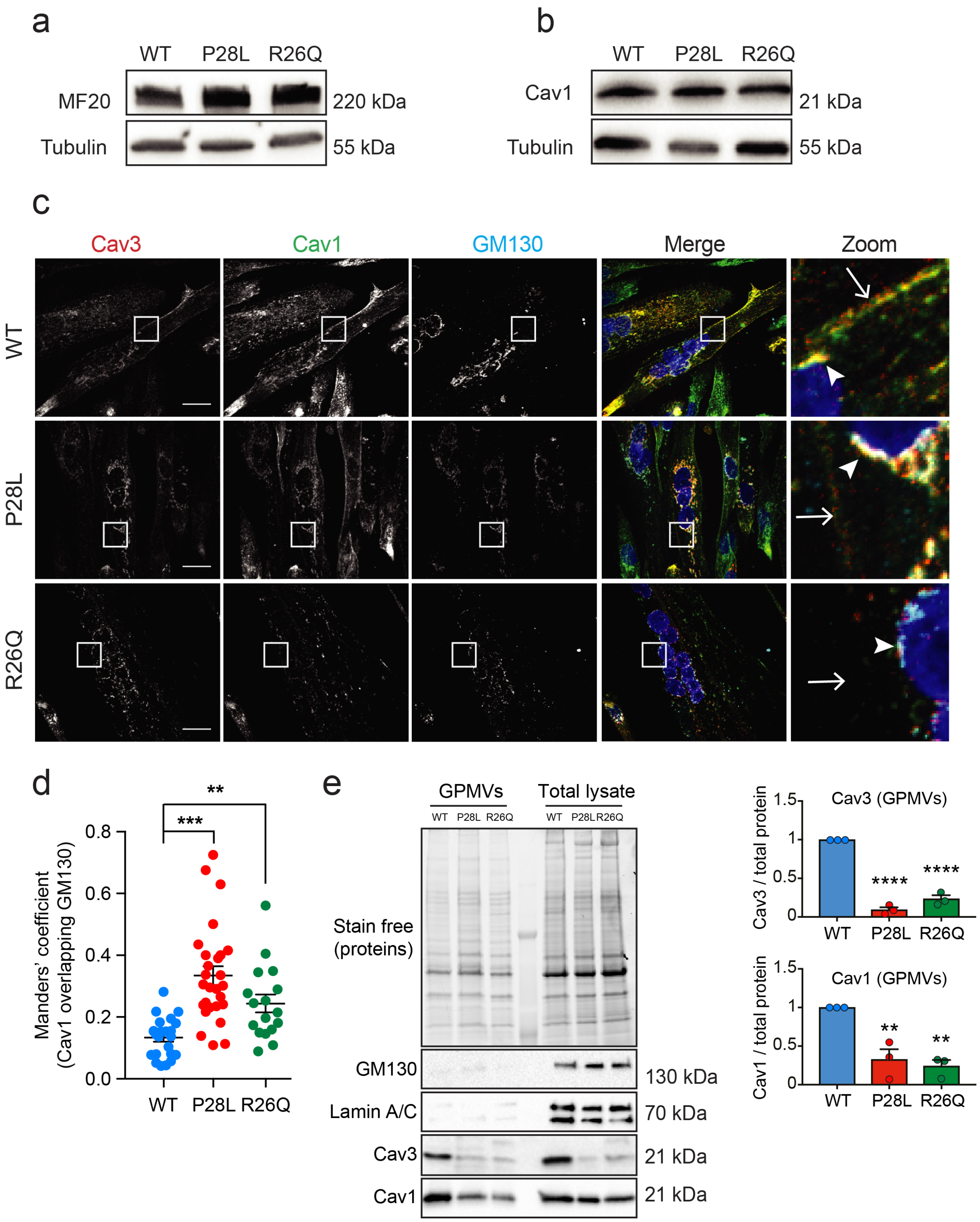

Supplementary Figure 1 | Cav1 expression in WT, Cav3 P28L and R26Q myotubes. (**a, b**) Immunoblot analysis of total levels of MF20 (myosin heavy chain) (**a**) and Cav1 (**b**) in WT, Cav3 P28L and Cav3 R26Q myotubes. Tubulin serves as a loading control. (**c**) Cav3 (red), Cav1 (green) and the Golgi marker GM130 (light blue) immunofluorescence were analyzed by confocal microscopy in WT, Cav3 P28L and Cav3 R26Q myotubes. Cav3 and GM130 staining are shown in Fig. 1d. (**d**) Quantification of colocalization in (c) expressed as the Mander's coefficient, indicating the proportion of Cav1 pixels containing the Golgi

apparatus marker GM130 pixels. (**e**) Immunoblot analysis and quantification of Cav3 and Cav1 levels in the plasma membrane fraction (GPMVs) and in the total cell lysate of WT, Cav3 P28L and Cav3 R26Q myotubes. GM130 and lamin A/C serve as controls for the Golgi apparatus, and nuclei respectively. Stain-free staining serves as loading control. (**c**) Scale bar = 10 µm. Reproducibility of experiments: (**a**), (**b**), (**c**) and (**e**) Representative data for 3 experiments. (**d, e**) Quantification was done on 3 independent experiments (for (**d**) WT n = 24 cells, P28L n = 27 cells, R26Q n = 17 cells). Mean value ± SEM. Statistical analyses were done using one-way ANOVA, ** P<0,01; *** P<0,0001; **** P<0,00001.

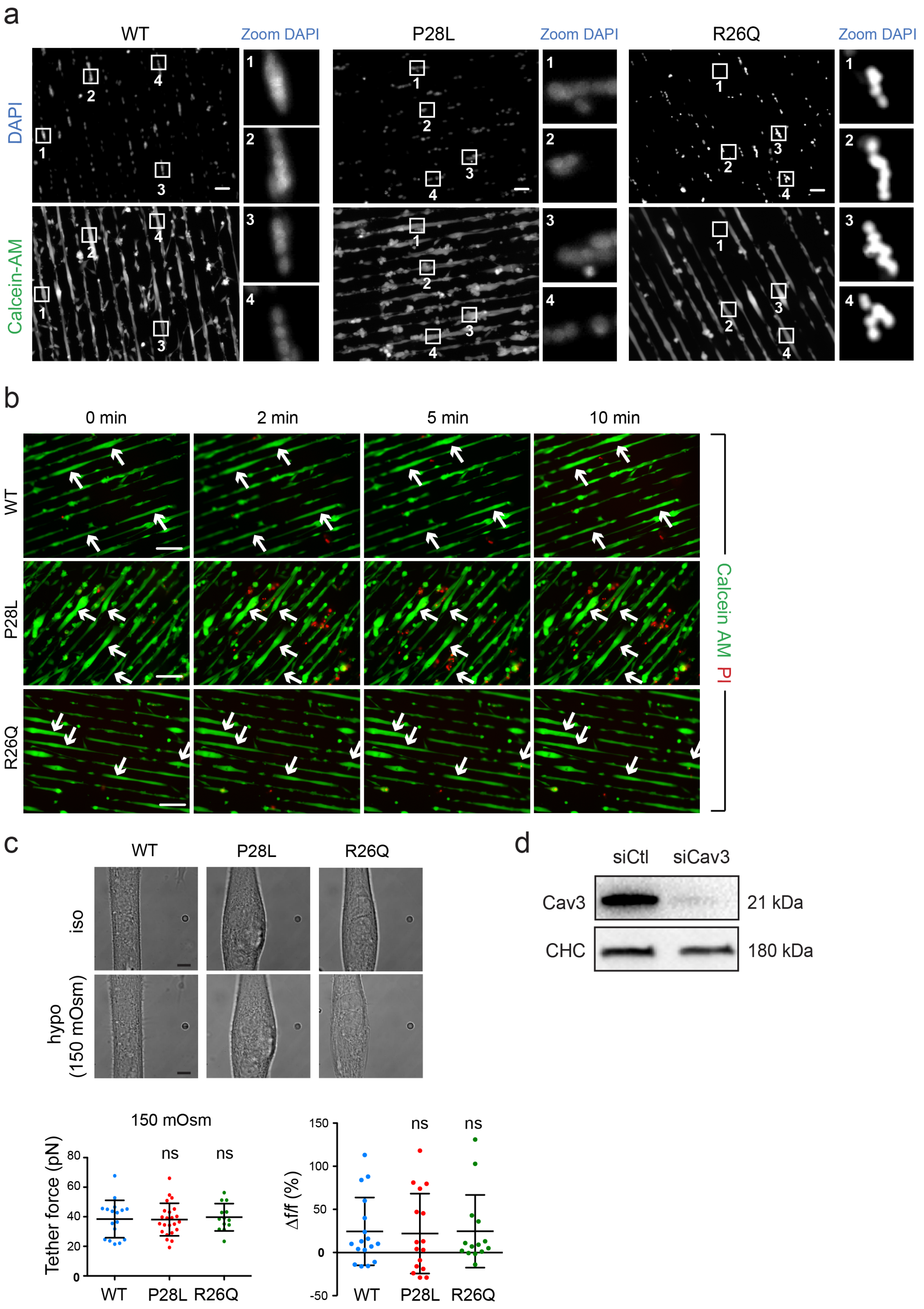

Supplementary Figure 2 | Efficient membrane tension buffering and mechanoprotection in Cav3 mutant myotubes under mild hypo-osmotic shock.

(**a**) Calcein-AM and DAPI fluorescence of WT, Cav3 P28L and Cav3 R26Q myotubes prior to hypo-osmotic shock in the membrane bursting assay described in **Figure 2c**. Insets show DAPI in myotubes indicated with arrows in **Figure 2c**. (**b**) Micropatterned WT, Cav3 P28L and Cav3 R26Q myotubes were loaded with calcein-AM (green). The medium was switched to a 150 mOsm medium supplemented with propidium iodide (PI, red). Representative pictures were taken at the indicated times during hypo-osmotic shock. Arrows correspond to myotubes and asterisks correspond to burst myotubes. (**c**) Membrane tension measurement analysis using optical tweezers and nanotube pulling on micropatterned WT, Cav3 P28L and Cav3 R26Q myotubes. Membrane tethers were pulled in the perpendicular axis of myotubes after micropatterning in resting conditions and 5 min after a 150 mOsm hypo-osmotic shock (upper panel). Membrane tension was analyzed in resting condition (lower panel, left) and the difference of membrane tension before and after hypo-osmotic shock was calculated, reflecting the percentage of increase of membrane tension upon mechanical stress (lower panel, right) (**d**) Immunoblot analysis of Cav3 depletion in **Figure 2e**. (**a, b**) Scale bar = 120 µm. (**c**) Scale bar = 5µm Reproducibility of experiments: (**c**) Quantifications were done on 5 independent experiments (WT n=17 cells, P28L n=16 cells, R26Q n=14 cells). Mean value ± SD. Statistical analyses were done using Kruskal-Wallis test; ns, non significant.

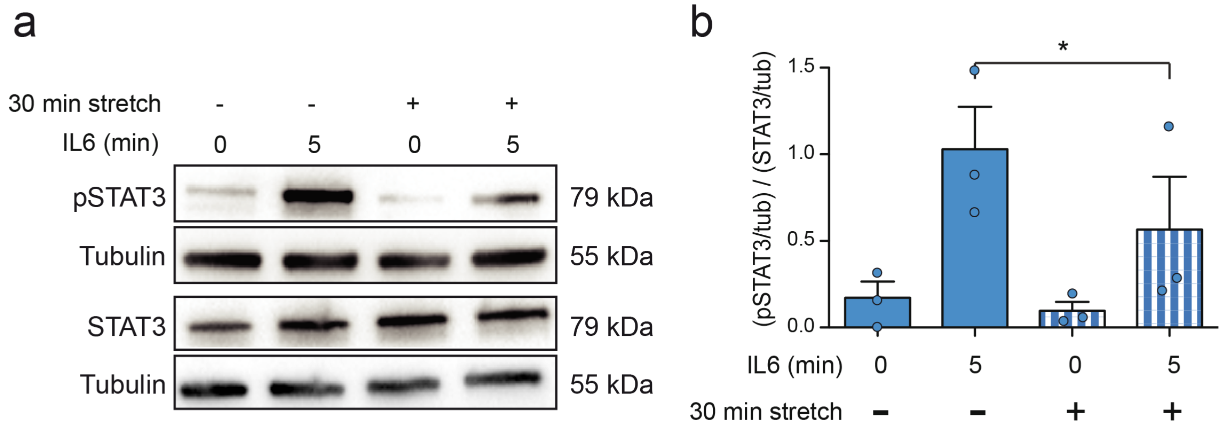

Supplementary Figure 3 | IL6/STAT3 signaling in WT myotubes under cyclic stretch.
(**a**) Immunoblot analysis of pSTAT3 and STAT3 levels in WT myotubes subjected or not to 30 min cyclic stretch. Myotubes were then stimulated or not with 10 ng mL^{-1} IL6 for 5 min. Tubulin serves as a loading control. (**b**) Quantification of STAT3 activation in (**a**) corresponding to the ratio pSTAT3 on STAT3 total levels after normalization to tubulin levels. Reproducibility of experiments: (**b**) Quantification was done on 3 experiments. Mean value ± SEM. Statistical analyses were done using two-tailed paired t test; * P<0,05.

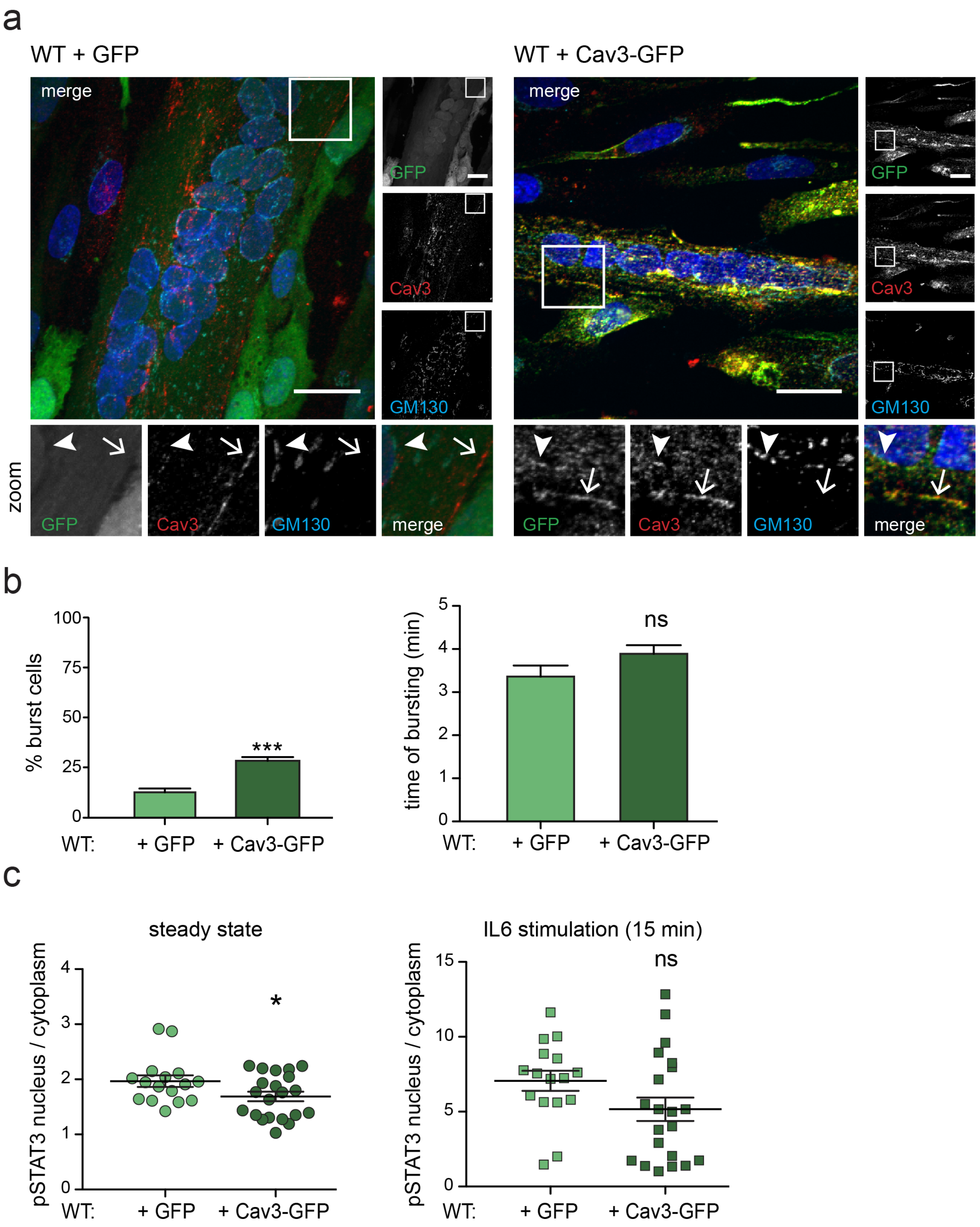

Supplementary Figure 4 | Effect of Cav3 expression in IL6 signaling in WT myotubes. (**a**) Immunofluorescent labeling of Cav3 and Golgi marker GM130 in WT GFP and WT Cav3-GFP transduced myotubes analyzed by confocal microscopy. Arrows and arrowheads in inset indicate the plasma membrane and the Golgi complex respectively. (**b**) Quantification of the percentage of burst myotubes after a 30 mOsm hypo-osmotic shock (left panel) and mean time of bursting in minutes (right panel) in (**a**). (**c**) Quantification of pSTAT3 nuclear translocation in WT GFP or WT Cav3-GFP transduced myotubes stimulated or not for 15 min

with 10 ng mL^{-1} IL6, corresponding to nuclei/cytoplasm mean intensity ratio of pSTAT3. (**a**) Scale bar = 10 µm. Reproducibility of experiments: (**a**) Representative data from 3 independent experiments. (**b**) Quantification was done on 3 independent experiments (% burst: GFP n=714 cells, Cav3-GFP n=610 cells; time of burst: GFP n=80 cells, Cav3-GFP n=171 cells). (**c**) Quantification was done on 3 independent experiments (control: GFP n=16 cells, Cav3-GFP n=21 cells; 15 min: GFP n=16 cells, Cav3-GFP n=21 cells). (**b, c**) Statistical analyses were done using two-tailed unpaired t test; ***P<0,0001; *P<0,05; ns, non significant.

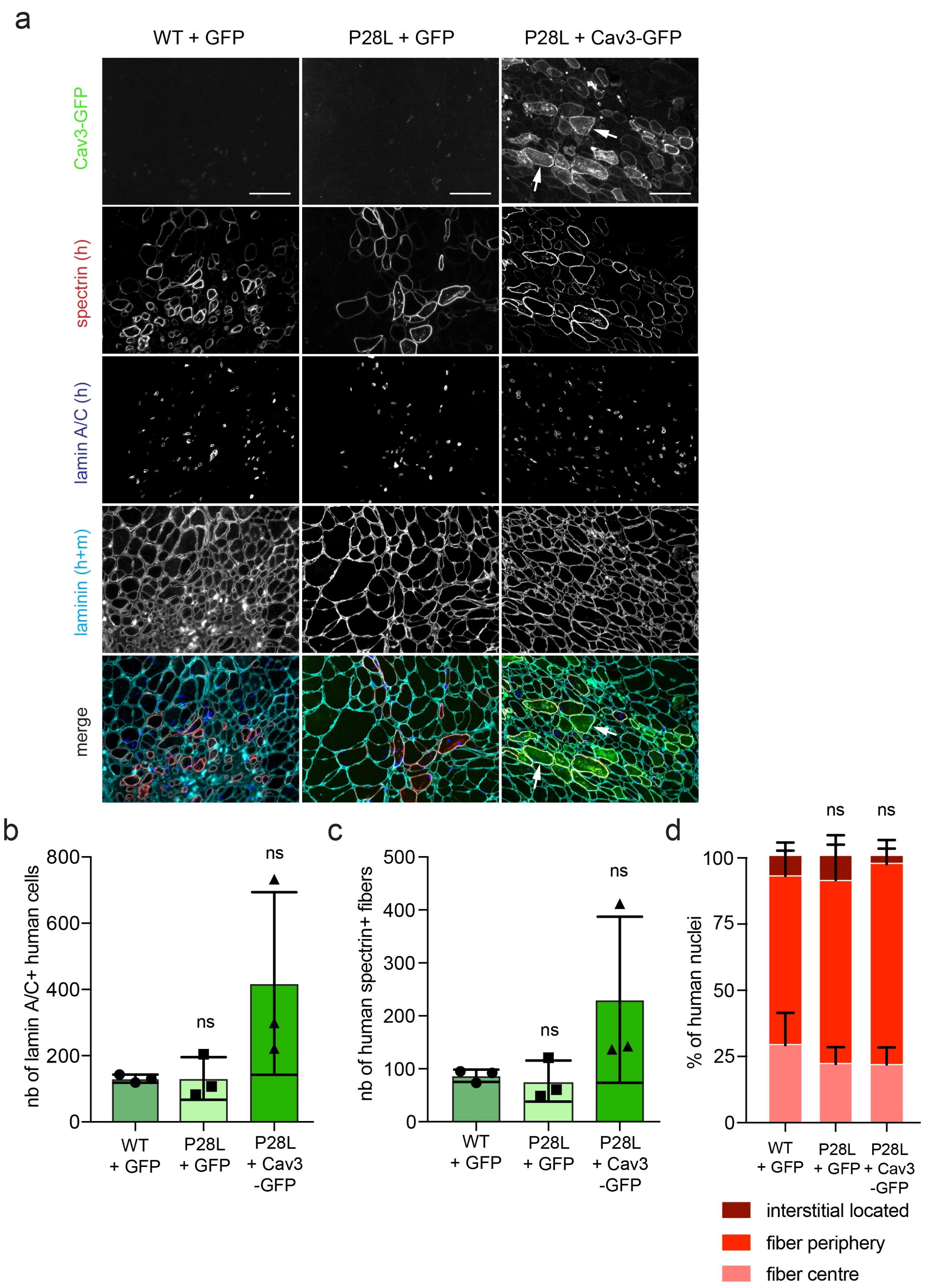

Supplementary Figure 5 | Characterization of transduced myoblasts injected into mouse muscle.

(**a**) Human nuclei of WT GFP, P28L GFP or P28L Cav3-GFP transduced myoblasts were visualized using an anti-spectrin and an anti-lamin A/C antibody. Fibers expressing human proteins were visualized using an anti-human spectrin-specific antibody. Fibers were visualized using and anti-laminin-specific antibody. Arrows indicate Cav3-GFP at the plasma membrane. (**b**) Quantification of the number of human cells positive for human specific lamin A/C staining in (**a**). (**c**) Quantification of the number of fibers containing cells positive for human specific spectrin staining in (**a**). (**d**) Quantification of the percentage of human nuclei in the interstitial area, at the periphery of the fibers or at the center of the fibers in (**a**). (**a**) Scale bar = 100 μm. Reproducibility of experiments: (**a**) Representative data of 3 independent experiments. (**b**, **c**, **d**) Quantification was done on 3 independent experiments. (**b**, **c**, **d**) Mean value ± SD. Statistical analysis was done using a one-way ANOVA analysis. ns, non significant.

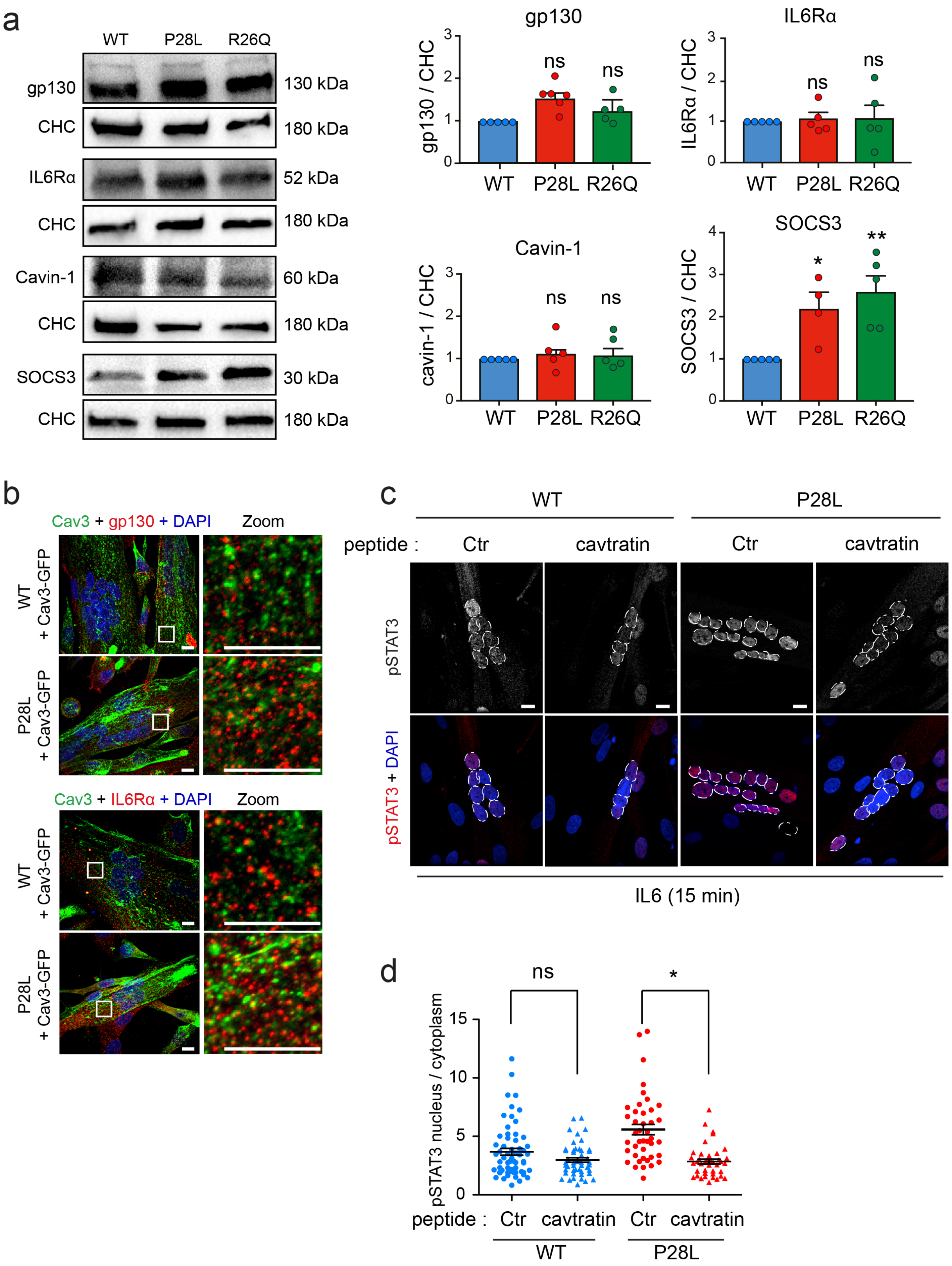

Supplementary Figure 6 | Inhibition of the IL6/STAT3 pathway involves the CSD.
(**a**) Immunoblot analysis and quantification of total levels of gp130, IL6Rα, Cavin-1 and SOCS3 in WT, Cav3 P28L and Cav3 R26Q myotubes. Clathrin heavy chain (CHC) serves as a loading control. (**b**) Immunofluorescent labeling of gp130 (red, upper panel), IL6Rα (red, lower panel) in WT Cav3-GFP and P28L Cav3-GFP transduced myotubes analyzed by

confocal microscopy. (**c**) Confocal microscopy of immunofluorescent labeling of pSTAT3 in WT and Cav3 P28L myotubes treated or not with a control peptide (Ctr) or with cavtratin (Caveolin Scaffolding Domain, CSD) for 4h, followed by stimulation or not with 10 ng mL^{-1} IL6 for 15 min. White dashed lines outline nucleus boundaries. (**e**) Quantification of pSTAT3 nuclear translocation in (**d**) corresponding to the ratio between the nuclei and the cytoplasm of the mean pSTAT3 intensity. (**b, c, d**) Scale bar = 10 µm. Reproducibility of experiments: (**a**) Representative data and quantification done on 5 experiments (**b**) Representative data out of 2 independent experiments. (**d**) Quantification was done on 3 independent experiments (WT: Ctr n = 61 cells, cavtratin n = 48 cells; P28L: Ctr n = 42 cells, cavtratin n = 40 cells). Mean value ± SEM. (**a, d**) Statistical analysis with one-way ANOVA analysis; * P<0,05; ** P<0,01; ns, non significant.

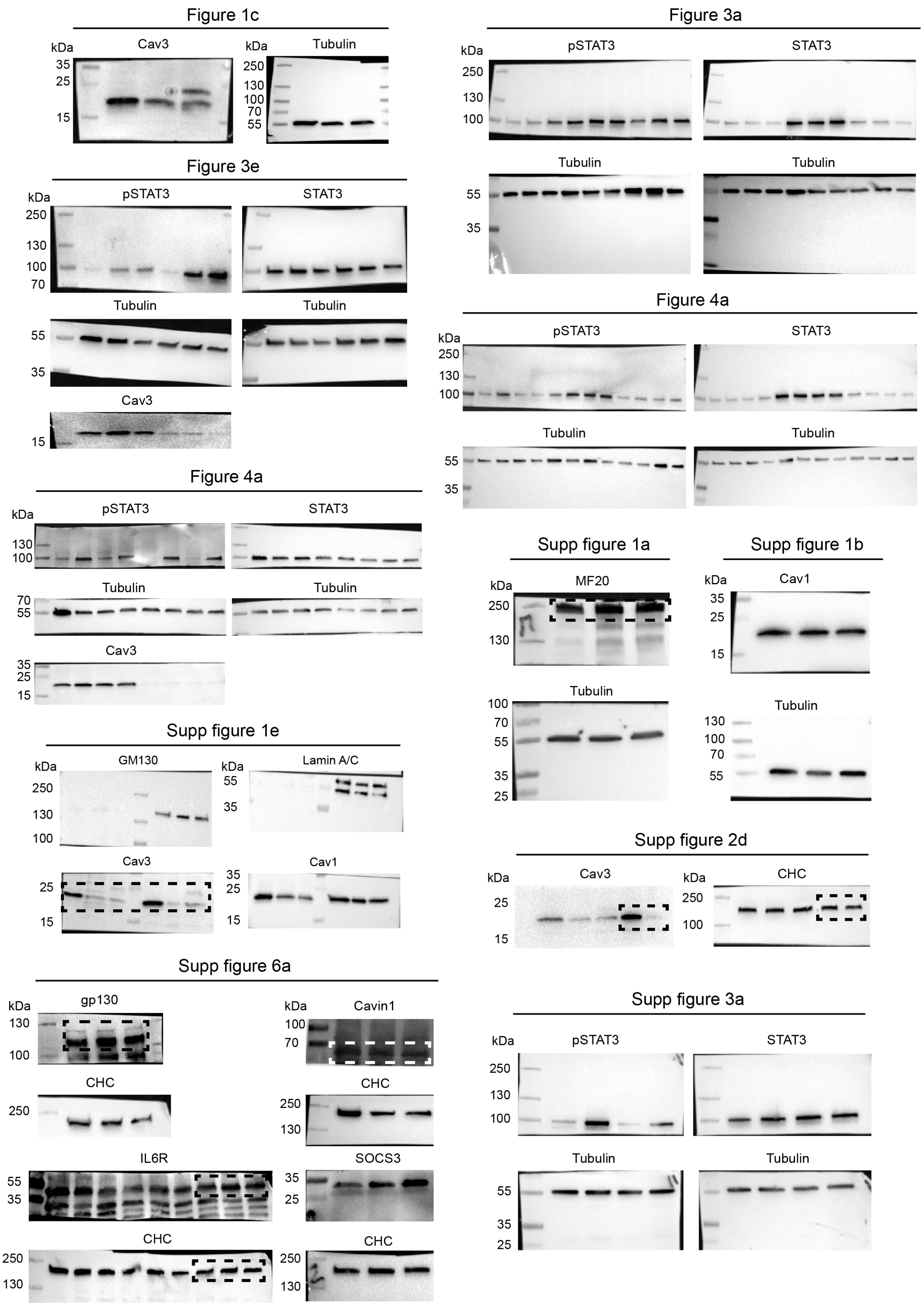

Supplementary Figure 7: Full scans of western blots.

Gene	siRNA sequence
CAV3	SI03068730 - CAGCTTTGACGGCGTGTGGAA<br>SI02625665 - TTGCGTTCACTTGTACTGTAA<br>SI02625658 - TAGGAAGGTGGCTCCAGTAAA<br>SI00146188 - CACCTTCACTGTCTCCAAGTA
Control	1022076 - ATTCTCCGAACGTGTCACGT
Gene	TaqMan gene expression assay ID
GAPDH	Hs02786624_g1
ACTC1	Hs01109515_m1
MYH8	Hs00267293_m1
SOCS3	Hs02330328_s1
ACTN2	Hs00153809_m1

Supplementary Table 1: siRNA sequences and qRT-PCR assays.

Supplementary Methods

Giant Plasma Membrane Vesicles. Cells were washed once with PBS and once with GPMV buffer (10 mM HEPES pH 7.4, 150 mM NaCl, 2 mM $CaCl_2$). Cells were subsequently incubated with GPMV buffer supplemented with 12 mM NEM and proteinase inhibitors cocktail for 1 hour at 37°C. GPMVs were decanted, and the suspension was centrifuged at 200g for 5 min at 4°C to remove cell debris. The supernatant was centrifuged at 100,000g for 1 hour at 4°C, and the GPMV-containing pellet was resuspended in PBS containing proteinase inhibitors. The total protein concentration was measured using Bradford assay. Samples were analyzed by Western Blot.

Animals. 2- to 3-month-old immunodeficient *Rag2*$^{-/-}$ *Il2rb*$^{-/-}$ mice were used as recipients for human cell implantation. Mice were anesthetized with an intraperitoneal injection of ketamine hydrochloride (80 mg kg^{-1}) and xylazine (10 mg kg^{-1}) (Sigma-Aldrich, St Louis, MO). This study was carried out in strict accordance with the legal regulations in France and according to European Union ethical guidelines for animal research. The protocol was approved by the Committee on the Ethics of Animal Experiments Charles Darwin N°5 (Protocol Number: 16903-2018092717023720). All surgery was performed under ketamine hydrochloride and xylasine anesthesia, and all efforts were made to minimize suffering. To induce severe muscle damage and trigger regeneration, the recipient *tibialis anterior* (TA) muscles were exposed to cryodamage, and a single injection of immortalized human cells (15 µl of cell suspension containing 5.10^5 cells in PBS) was administered using a 25-µl Hamilton syringe, in a single midpoint site along the longitudinal axis of the TA. The skin was then closed with a fine suture[1]. Three weeks after transplantation, the recipient TA muscles were dissected, mounted in gum tragacanth (6% in water; Sigma-Aldrich), and frozen in liquid nitrogen-cooled isopentane for later analysis.

Immunofluorescence. For immunofluorescence, 5 µm transverse cryostat sections were used either unfixed (for lamin A/C and spectrin) or fixed with 4% PFA for 5 minutes at room temperature, washed twice in PBS and blocked with 4% bovine serum albumin/PBS. Sections were incubated with primary antibody for 1 hour at room temperature, then washed in PBS and stained with appropriate secondary antibodies for 45 minutes at room temperature[1]. Antibodies used were directed against lamin A/C (clone JOL2, mouse IgG1, 1:300; AbCam, Cambridge, Cambridgeshire, UK), spectrin (NCL-Spec1, clone RBC2/3D5, mouse IgG2b,

1:50; Novocastra, Newcastle-upon-Tyne, Tyne and Wear, UK), and laminin (rabbit polyclonal, Z 0097, 1:400; Dako, Trappes, France). Images were visualized using a microscope (Olympus Corp., Tokyo, Japan), and digitized using a charge-coupled device (CCD) camera (Olympus Corp., Tokyo, Japan).

CSD mimicking peptides. Peptides corresponding to the full-length (amino acids 82-101; DGIWKASFTTFTVTKYWFYR, cavtratin) or modified (amino acids 82-101; WGIDKASFTTFTVTKYWFRY, Ctr) scaffolding domain of Cav3 were synthesized as a fusion peptide to the C-terminus of the *Antennapedia* internalization sequence (RQIKIWFQNRRMKWKK) by Biomatik (Wilmington, USA). Cells were treated for 4 hours with 1μM CSD mimicking peptides resuspended in serum free medium 0,2% BSA (v/v).

Supplementary References

1. Negroni, E. et al. *In vivo* myogenic potential of human CD133+ muscle-derived stem cells: a quantitative study. *Mol Ther* **17,** 1771–1778 (2009).
